# Mitochondrial DNA mutations in Korean patients with Leber’s hereditary optic neuropathy

**DOI:** 10.1038/s41598-024-56215-x

**Published:** 2024-03-08

**Authors:** Hee Kyung Yang, Moon-Woo Seong, Jeong-Min Hwang

**Affiliations:** 1 Department of Ophthalmology, Seoul National University College of Medicine, Seoul National University Bundang Hospital, 82 Gumi-ro, 173 Beon-gil, Bundang-gu, Seongnam, Gyeonggi-do 13620 Republic of Korea; 2 Department of Laboratory Medicine, Seoul National University College of Medicine, Seoul National University Hospital, Seoul, Republic of Korea

**Keywords:** Leber’s hereditary optic neuropathy, Mitochondrial DNA mutation, Spectrum, Koreans, Eye diseases, Optic nerve diseases

## Abstract

In order to explore the spectrum of mitochondrial DNA (mtDNA) mutations in Korean patients with Leber's hereditary optic neuropathy (LHON), we investigated the spectrum of mtDNA mutations in 145 Korean probands confirmed with the diagnosis of LHON. Total genomic DNA was isolated from the peripheral blood leukocytes of the patients with suspected LHON, and mtDNA mutations were identified by direct sequencing. Analysis of mtDNA mutations revealed seven primary LHON mutations including the nucleotide positions (nps) 11778A (101 probands, 69.2%), 14484C (31 probands, 21.2%), 3460A (5 probands, 3.4%), and G3635A, G3733A, C4171A, and G13051A mutations in one proband each. In addition, two provisional mtDNA mutations at nps T3472C, and G13259A were each found in one proband, respectively. Another provisional mtDNA mutation at np T3394C was found in two probands. In conclusion, the spectrum of mtDNA mutations in Korean patients with LHON may differ from other ethnicities, which is characterized by high prevalence of 11778A and 14484C mutations, and a low prevalence of the 3460A mutation.

## Introduction

Leber’s hereditary optic neuropathy (LHON) is a mitochondrial optic neuropathy, characterized by loss of central vision^1^. Among more than 20 kinds of mitochondrial DNA (mtDNA) mutations (http://www.mitomap.org/), over 90% of LHON pedigrees harbored one of the three primary mtDNA mutations at nucleotide positions (nps) C3460A, G11778A, and T14484C^1,2^. Prognosis could be different according to the type of mtDNA mutation in LHON^1,3,4^, therefore, identification of the primary mtDNA mutation is important both for the probands as well as their family.

The prevalence of mtDNA mutation varies considerably according to ethnicity.^2,5–19^ We once performed a study to investigate the presence of 14 primary mutations and one secondary mutation in 82 unrelated probands suspected to have LHON,^18^ and found only three major primary mutations in 60 (73%) of the 82 probands, and no other mutations. Among the 60 probands with confirmed LHON mutation, 11778A mutation was most commonly found in 46 probands (77%), followed by 14484C in 13 (22%), and 3460A in one proband (1%), which was quite different from the spectrum in Caucasians^2,5–9^ as well as from that in other Asians^10–17^. In addition, we have reported two provisional mtDNA mutations such as T3472C^20^ and G13259A^21^. Herein, we performed this study to further identify the relative frequencies of mtDNA mutations among confirmed LHON mutations in Asian patients.

## Results

Of 145 probands, the three major primary mtDNA mutations were identified in 137 probands, in the order of G11778A, T14484C, and C3460A in 101 (69. 6%), 31 (21. 4%), and 5 (3.4%) probands, respectively (Table 1,2). Other primary mutations at nps G3635A, G3733A, C4171A, and G13051A were found in one proband each (Tables 1,2). In addition, the previously reported two provisional mtDNA mutations at nps T3472C^20^, and G13259A^21^ were found in one proband each. Another provisional mtDNA mutation at np T3394C was found in two probands. T3394C was homoplasmic, and T3472C and G13259A were heteroplasmic (Supplementary Table 1).

**Table 1:** Analysis of mitochondrial DNA mutations in 145 Korean probands with Leber’s hereditary optic neuropathy.

	Gene locus	Mutation	No. of probands (%)
Primary	MT-ND1	C3460A	5 (3.4)
	MT-ND1	G3635A^28^	1 (0.7)
	MT-ND1	G3733A^29^	1 (0.7)
	MT-ND1	C4171A^3^	1 (0.7)
	MT-ND4	G11778A	101 (69.6)
	MT-ND5	G13051A^30^	1 (0.7)
	MT-ND6	T14484C	31 (21.4)
Secondary	MT-ND1	T3394C^23^	2 (1.4)
	MT-ND1	T3472C^20^	1 (0.7)
	MT-ND5	G13259A^21^	1 (0.7)
Total			145

*MT-ND* mitochondrial encoded NADH dehydrogenase.

**Table 2:** Relative frequency of the three primary mutations confirmed among different populations.

Population	G11778A (%)	T14484C (%)	G3460A (%)	Other primary mutations
Northern Europe, UK, Australia^2^	69	14	13	
Finland^5^	79	4	17	
Denmark^6^	67	18	13	A3395G
France^7^	72	12	16	
Italian^8^	66	16	18	
The US^9^	49	23	28	
India (North)^10^	100*	9*	Nil	
India (South)^11^	90	7	Nil	C4171A T12338C
India^12^	85	13	2	
China (Han)^13^	86	13	1	
Taiwan^14^	92	8	Nil	
Japan^15^	87	9	4	
Japan^16^	87	11	2	
Japan^17^	82	15	Nil	G11696A T12811C
Korea^18^	76	22	2	
Korea^19^	87	13	Nil	
Present study	69	21	3	G3635A G3733A C4171A G13051A

*N/T* not tested.

*One family with both 11778A & 14484C mutation.

## Discussion

In this study, we found seven primary mutations of LHON: Three major primary mutations of 11778A, 14484C and 3460A in 69.2%, 21.4%, and 3.4%, respectively, and 4 additional primary mutations of G3635A, G3733A, C4171A, and G13051A in one proband each. In addition, two provisional mtDNA mutations at nps T3472C and G13259A were each found in one proband, respectively. Another provisional mtDNA mutation at np T3394C was found in two probands. The 11778A mutation is the most common type of LHON mutation worldwide except for the French Canadians. The frequency of 11778A mutation varies among different ethnic groups (Table 2). In Caucasians, 11778A mutation was reported in 49 to 82%.^2,5–9^. In Asians, 11778A mutation was found in 85 to 100% of Indian LHON pedigrees,^10–12^ 86 to 92% of Chinese,^13,14^ and 82 to 87% of Japanese^15–17,22^. In Koreans, two previous reports reported 11778A mutation in 76 to 87%^18,19^. Meanwhile, in the present study, we found 11778A mutation in 69% which is the lowest reported incidence among Asian pedigrees.

The frequency of 14484C mutation also differs according to ethnicity, 4 to 23% in Caucasian LHON pedigrees^2,5–9,23^, 7 to 13% in Indians,^10–12^ 8 to 13% in the Chinese^13,14^ and 9 to 15% in the Japanese^15–17,22^. In Koreans, it was the second most common type of LHON mutation with an incidence of 13^19^ to 22%^18^, and 21% in this report. Thus, the 14484C mutation is relatively common in Koreans, and its reported frequency is the second highest among all ethnicities.

The presence and frequency of 3460A mutation may show the most profound ethnic difference in LHON mutation. The 3460A mutation is the least common among the three major primary mutations in Asians, but more common than 14484C in Finnish^5^ and the United States^9^. It has been found in 13 to 28% of Caucasians in North America and Europe^2,5–9,24^, but very rare in Asians. Two reports from Indians,^10,11^ and one report from Chinese,^14^ Japanese^17^ and Korean pedigrees^19^ did not find any patients with 3460A mutation. In Koreans, a previous report found 3460A mutation in 2%^18^, which is comparable to this study of 3%.

The overall spectrum of mtDNA mutations has been reported in various studies. In Japanese patients with LHON, Mashima et al^15^ found mtDNA mutations at np 3460 in 4% (3/68 patients), 11778 in 87% (59/68), and 14484 in 9% (6/68). In addition, they identified 5 secondary mtDNA mutations at np 3394, 4216, 7444, 9438 or 13708 in 15% (10/68) (Table 2). There were no differences in terms of clinical features of the Japanese LHON patients harboring the three primary LHON mutations compared to Caucasian patients. Matsumoto et al^25^ reported mtDNA mutations at np 3394 (1 patient), 3316 (2), 3496 (1), 3497 (2), and 13708 (1) in 19 Japanese LHON patients with three primary mutations. They suggested that the mutations at np 3316, 3496, and 3497 are secondary mutations of LHON^25^. Later, Ueda et al^16^ performed a nationwide survey and reported 38 patients (86%) with the 11778A mutation, 5 (11%) with 14484C mutation, and 1 (2%) with 3460A mutation (Table 2). The second nationwide survey in 2019^17^ found 11778A mutation in 82% (45/55 patients), 14484C in 29% (8/55), and no patients with 3460A mutation (Table 2). They found two other mutations at np 11696 (1/55) and 11281 (1/55). In addition, there exists a male predominance and lower percentage of familial LHON cases harboring the 3460A or 14484C mutations indicating homogeneity of the mtDNA mutation in Japanese LHON pedigrees^22,26^. Regarding other Asian LHON studies, two reports of Han Chinese LHON pedigrees found 86 to 92% of 11778A, 8 to 13% of 14484C, and 0 to 1% of 3460A mutations^13,27^ (Table 2). Indian LHON pedigrees showed 85 to 100% of 11778A, 7 to 13% of 14484C, and 0 to 2% of 3460A mutations^10–12^. In short, Asian LHON pedigrees showed significantly higher frequencies of 11778A mutation than in Caucasians (80–92% vs 67–82%), and a lower prevalence of 3460A mutation (0–4 vs 13–18%) (Table 2). In this report, Korean LHON pedigrees showed the lowest frequency of 11778A mutation (69%) among Asian LHON pedigrees, the second highest of 14484C mutation (21%) worldwide, and a low frequency of 3460A mutation (3%) (Table 2).

In conclusion, the spectrum of mtDNA mutations in Korean patients with LHON is characterized by high prevalence of 11778A and 14484C mutations, and a low prevalence of the 3460A mutation, showing the difference from other ethnicities. Further studies to reveal unidentified pathogenic mitochondrial mutations may provide a better insight into the spectrum for LHON mutations in diverse ethnic groups.

## Patients and Methods

### Patients

A retrospective review of mtDNA analyses was performed on 145 consecutive pedigrees in whom mtDNA mutations associated with LHON were found at Seoul National University Bundang Hospital between 2003 and 2023. Informed consent for a genetic test was obtained from all of the patients and/or their legal guardians. The study protocol adhered to the tenets of the Declaration of Helsinki and was approved by the Institutional Review Board of Seoul National University Bundang Hospital (B-1908–558-005). A screening test was performed for the three major LHON primary mutations at nps 3460A/ND1, 11778A/ND4, and 14484C/ND6 in patients who fulfilled the following criteria of (1), (2) and one of (3a-c): (1) history of acute or subacute simultaneous or sequential bilateral optic neuropathy without ocular pain, (2) central scotoma, (3a) maternal history of optic neuropathy, (3b) telangiectatic microangiopathy of the optic disc, and (3c) no response to treatment with corticosteroids. All other causes of optic neuropathy were excluded by ophthalmological and neurological examinations. Some patients underwent additional whole mtDNA analyses as well as orbit and brain magnetic resonance imaging.

### DNA analyses

Total mtDNA was extracted from peripheral blood leukocytes using the standard method. Diagnostic restriction site gain or loss was confirmed by direct sequencing using Thermo Sequenase radio-labeled terminator cycle sequencing kits (Amersham, Buckinghamshire, UK) and 6% polyacrylamide sequencing gels.

### Supplementary Information

Supplementary Information.

## Data Availability

The Institutional Review Board of Seoul National University Bundang Hospital/Ethics committee has placed ethical restrictions to protect patient identities. However, the data are available to anyone who is interested without restriction. The minimal data set will be available upon request. For data requests, please contact the SNUBH IRB office at 82-31-787-8804, 98614@snubh.org.
