# Supplementary material for: Mitochondrial DNA mutations in Korean patients with Leber’s hereditary optic neuropathy
Source: Sci Rep. 2024 Mar 8;14:5702. doi: 10.1038/s41598-024-56215-x (PMC10923793; doi:10.1038/s41598-024-56215-x)
Supplement: Supplementary file 1 — Supplementary Information. [file 41598_2024_56215_MOESM1_ESM.docx]

Supplementary Table 1. Characteristics of four provisional LHON mutations

| Mutation | Gene | AA change | Homo/heteroplasmy | gnomAD^*^ | APOGEE^*^ | Mitomap [ClinVar] ^*^ | ACMG calssification^**^ | Evidence^**^ | Haplogroup^***^ |
| --- | --- | --- | --- | --- | --- | --- | --- | --- | --- |
| m.3394T>C | MT-ND1 | Y30H | Homoplasmy | 0.9% (514/56420) | P (0.76) | Reported [Conflicting] | VUS | PP3, BS1 | NA |
| m.3472T>C | MT-ND1 | F56L | Heteroplasmy | 0.0017% (1/56431) | N (0.46) | Reported [VUS] | VUS | PM2_supporting | B4 |
| m.4216T>C | MT-ND1 | Y304H | Homoplasmy | 11.8% (6570/55886) | P (0.73) | Reported [Conflicting] | Benign | PP3, BA1 | NA |
| m.13259G>A | MT-ND5 | S308T | Heteroplasmy | NA | P (0.72) | NA | VUS | PM2_supporting, PP3 | N9 |

* gnomAD, APOGEE and Mitomap [ClinVar] information was taken from Mitomap (https://www.mitomap.org/allelesearch.html)

** ACMG classification was done according to McCormick EM et al. (Hum Mutat. 2020;41(12):2028-2057. doi:10.1002/humu.24107)

*** Haplogroup was designated using Haplogrep3 (https://haplogrep.i-med.ac.at)

Three provisional LHON mutations except m.4216T>C were classified as VUS according to ACMG/AMP guidelines for mitochondrial DNA variant interpretation (Supplementary Table 1). m.4216T>C was classified as a benign variant because it was found in the normal population with relatively high frequency (>1%, BA1) and it was associated with haplogroup J and T, although it has been reported with conflicting interpretation of pathogenicity in ClinVar. m.3394T>C was also frequent in the normal population (0.5% - 0.99%, BS1) and it has been reported as a specific marker of some haplogroups, like M9a, J1c and C4a. However, m.3394T>C was predicted as having pathogenic impact in the in-silico prediction tool, APOGEE (PP3). Its clinical significance cannot be determined under these situations. The other two LHON missense mutations, m.3472T>C and m.13259G>A were rarely found (<0.002%) in the normal population, so PM2_supporting evidence was assigned to each mutation, respectively.
